# Cancer and embryo expression protein 65 promotes cancer cell growth and metastasis

**DOI:** 10.3892/ol.2015.2958

**Published:** 2015-02-12

**Authors:** GENGLIN JIN, LIRONG PENG, JIANZHI ZHANG, LIKE QU, CHENGCHAO SHOU

**Affiliations:** Key Laboratory of Carcinogenesis and Translational Research (Ministry of Education), Department of Biochemistry and Molecular Biology, Peking University Cancer Hospital and Institute, Beijing 100142, P.R. China

**Keywords:** cancer and embryo expression protein 65, growth, migration, invasion, metastasis

## Abstract

Cancer and embryo expression protein 65 (CEP65) is a centrosomal protein that is expressed at relatively high levels in embryonic tissue and different cancerous tissues, but its role in tumorigenesis remains unknown. In the present study, CEP65 was stably expressed in AGS gastric cancer cells. CEP65 was found to promote cell growth in the MTT assay and to enhance cell migration and invasion in Transwell chamber assays. To validate results from the *in vitro* experiments, CEP65 was stably expressed in BICR-H1 breast cancer cells through adenovirus-mediated transduction. By inoculating BICR-H1 cells on chick chorioallantoic membrane (CAM), it was found that CEP65 promotes cell growth on the CAM and increases cell metastasis to the lungs of the chicken. By utilizing a xenograft severe combined immunodeficiency mouse model, CEP65 was also found to accelerate BICR-H1 cell growth and metastasis to the lungs. Furthermore, it was shown that CEP65 increases matrix metalloproteinase (MMP)2 activity in zymographic assays, however, microarray screening and reverse transcription polymerase chain reaction validation revealed that CEP65 had no effect on the expression levels of *MMP2* or *MMP9*, but decreased the expression levels of metastasis-associated genes, *TIMP2*, *RAP* and *VTN*. Taken together, the results of the present study demonstrated the oncogenic function of CEP65 in promoting cancer cell growth and metastasis.

## Introduction

3H11 is a monoclonal antibody (mAb) derived from mice sequentially immunized with varying cancer cell lines (1). Immunohistochemical analysis has revealed that 3H11 mAb is able to bind to various tissues from liver, lung, bladder, breast, colon and gastric cancer (1). In addition, isotope-labeled 3H11 mAb has been used as a radioimaging reagent in clinical tumor imaging (2). The antigen recognized by 3H11 (3H11-Ag) has been previously cloned through cDNA library screening (3). A bioinformatics analysis has revealed that this novel gene (GenBank accession no., AF317887; located on chromosome 12q21) has 17 exons and encodes 589 amino acids. Furthermore, using northern blot analysis, a 2.3-kb transcript has been detected, which is widely expressed in embryonic and cancerous tissues, but not in adjacent normal tissues (3), indicating that its function may be associated with cancer development and progression. Based on its expression profile and theoretical molecular weight, we defined 3H11-Ag as cancer and embryo expression protein 65 (CEP65) (4).

Previous studies have demonstrated the presence of high homology between CEP65 and centrosomal protein nephrocystin-6 (NPHP6), which is a protein frequently mutated in several hereditary diseases (5). In addition, a previous study has investigated the nuclear distribution and revealed the potential centrosomal localization of CEP65 (6); thus, CEP65 represents a novel member of the centrosome-binding protein family. The centrosome functions as a centre for microtubule organization and is involved in cell growth, division, migration and polarity (7). Since deregulated expression of centrosomal proteins is closely associated with hereditary diseases and tumor development (5,7), CEP65 may be involved in the control of malignant phenotypes of cancer cells. The present study aimed to investigate whether CEP65 promotes cancer cell growth and invasiveness *in vitro* and *in vivo*. Furthermore, the effect of CEP65 on matrix metalloproteinase (MMP)2 activity and the expression levels of the metastasis-associated genes, *RAP*, vitronectin (*VTN)* and tissue inhibitor of metalloproteinases-2 (*TIMP-2*), were investigated to establish whether CEP65 is an oncogene and a potential target for cancer therapy.

## Materials and methods

### Cell culture

The human gastric cancer AGS cell line (American Type Culture Collection, Manassas, VA, USA) was cultured in Ham’s F-12 medium (Sigma-Aldrich, St. Louis, MO, USA) supplemented with 10% fetal bovine serum (FBS; Invitrogen Life Technologies, Carlsbad, CA, USA) in a humidified incubator containing 5% CO_2_ at 37°C. Human embryonic kidney 293 (HEK-293) and NIH3T3 cells were obtained from the American Type Culture Collection and cultured in Dulbecco’s modified Eagle’s medium (Bioroc Pharmaceutical & Biotech Co. Ltd., Tianjin, China) supplemented with 10% FBS. In addition, BICR-H1 breast cancer cells were provided by Dr Zhiqian Zhang (Peking University Cancer Hospital and Institute, Beijing, China) and cultured in RPMI 1640 medium (Invitrogen Life Technologies) supplemented with 10% FBS.

### Generation of stable cell lines expressing CEP65

AGS-CEP65 and AGS mock-transfected (AGS-M) cells were generated by transfecting pcDNA3-CEP65 and pcDNA3 alone, respectively, into AGS cells using Lipofectamine^®^ 2000 (Invitrogen Life Technologies). The transfected cells were subsequently selected for using 600 μg/ml G418 (Sigma-Aldrich). After three weeks, pooled colonies were recovered. Recombinant adenoviruses were generated using the AdEasy Adenoviral Vector system (Agilent Technologies, Santa Clara, CA, USA), as previously described (8). Briefly, full-length CEP65 cDNA and antisense-CEP65 cDNA were cloned into the AdEasy Shuttle vector, pAdTrack-cytomegalovirus. The resultant plasmids were linearized and co-transformed into *E. coli* BJ5183 cells (American Type Culture Collection) with an adenoviral backbone plasmid, pAdEasy-1. Subsequently, the recombinants were selected for with kanamycin. The linearized plasmids were transfected into HEK-293 cells using Lipofectamine^®^ 2000 to yield the pAd-CEP65(+) and pAd-CEP65(−) viruses. The cells were infected using a multiplicity of infection (MOI) value of 10. The expression of exogenous CEP65 was detected using reverse transcription polymerase chain reaction (RT-PCR) and western blot analysis.

### Cell proliferation assay

In total, 100 μl of sample (2×10^4^ cells) was seeded in 96-well plates, using three replicates for each cell line. The cells were harvested at 12, 24, 36, 48 and 60 h after seeding, and the proliferation rate was measured using an MTT assay kit from Promega Corporation (Madison, WI, USA). The absorbance [optical density (OD) values] was measured at 492 nm using a microplate reader (iMark; Bio-Rad Laboratories, Inc., Richmond, CA, USA).

### Cell adhesion assay

To investigate cell adhesion, 96-well microplates were coated with 6 μg Matrigel/well (Collaborative Biomedical Products, Atlanta, GA, USA) and allowed to dry in a laminar flow cabinet overnight at room temperature. Subsequent to being washed three times with PBS, the wells were blocked with 30 μl solution, containing 20 mg/l bovine serum albumin (Sigma-Aldrich) in F-12 medium, for 1 h at 37°C. Subsequently, 8×10^4^ cells (100 μl; in F-12 medium) were seeded into the blocked wells and allowed to adhere for 1 h at 37°C. Following incubation, the wells were washed three times with PBS and the number of remaining cells was determined by an MTT assay.

### Cell migration and invasion assays

A cell migration assay was performed using tissue culture-treated 6.5-mm Transwell chambers with 8.0-μm pore membranes, which were obtained from Corning Incorporated (Corning, NY, USA). The bottom surfaces of the membranes were coated with 20% FBS in F12 medium by incubation for 2 h at 37°C. Conditioned medium was collected from a NIH3T3 cell culture and added to the bottom chambers (800 μl/chamber) along with 50 μg/ml fibronectin. Next, 2.5×10^4^ single AGS cells in serum-free F-12 medium (500 μl) were added to the top chamber of each Transwell. The cells were allowed to migrate for 12 h at 37°C in an atmosphere containing 5% CO_2_. Following removal of the remaining cells from the top chamber, a cotton swab was used to clean the top surface of each membrane. Subsequently, the cells penetrating through to the bottom surface of the membrane were fixed in methanol. The filters were stained in hematoxylin for 10 min, and the cells on the lower surface of the filter were counted in five randomly selected fields under a light microscope (Eclipse 80i; Nikon Corporation, Tokyo, Japan) (magnification, ×200). The invasion assay was performed using a procedure similar to the aforementioned migration assay procedure, with the exception that the upper surface of the membrane was coated with Matrigel and the cells were allowed to invade for 16 h at 37°C.

### In vivo cancer cell growth and metastasis assays

All animal experiments involving SCID mice were approved by the Medical Ethics Committee of the Peking University Cancer Hospital and Institute, and all procedures were performed in accordance with the animal welfare guidelines of the National Insitute of Health (NIH Publication No. 85-23; revised 1985). To investigate metastasis, chick chorioallantoic membrane (CAM) and severe combined immunodeficiency (SCID) mouse model assays were conducted as previously described (9), with minor modifications. Briefly, for the CAM assay, BICR-H1 cells were infected with pAd, pAd-Cep65(+) or pAd-Cep65(−). After 24 h of infection, the cells were harvested and added onto the CAM of 10-day-old chicken embryos of the white leghorn chicken (*Gallus gallus domesticus* L.; obtained from China Agricultural University, Beijing, China). A window was cut in the egg shell, which was sealed with Durapore tape following addition of the cancer cells onto the CAM. The eggs were incubated at 37.8°C and 80% relative humidity. After seven days, the tumor weight was measured and the lungs of the chicken embryos were checked using a fluorescence microscope (TiU; Nikon Corporation). Embyos were sacrificed using 30 min of 90% CO_2_ treatment. The cells in each sample were quantified from 20 random visual fields (x200) under a fluorescence microscope.

For the SCID mice model assay, infected BICR-H1 cells (4×10^6^ for each) were injected into the mammary fat pad of five-week-old female SCID mice (Weitong Lihua Experimental Animal Technology Co., Ltd., Beijing, China). After eight weeks, the mice were sacrificed by euthanasia and the tumor weight was measured. The lungs of the SCID mice were removed and analyzed by hematoxylin and eosin staining on fresh-frozen sections.

### Zymographic assay

A zymographic assay was performed according to the procedure described by Wang *et al* (10), with minor modifications. Briefly, conditioned F-12 medium was obtained from 24-h serum-deprived cells at 70–80% confluence and concentrated by 20-fold. Concentrated medium of 2×10^5^ cells was electrophoresed using 10% SDS-PAGE containing 0.1% gelatin, and incubated for 24 h in freshly prepared developing buffer [50 mM Tris-HCl (pH 8.0), 50 mM CaCl_2_ and 0.02% NaN_3_], following removal of the SDS. The degree of digestion, which was assessed by the density of the bands following staining and destaining, was proportional to the enzymatic activity.

### Western blot analysis

Cells were harvested in lysis buffer, containing 50 mM Tris-HCl (pH 7.4), 250 mM NaCl, 2 mM EDTA, 1% SDS, 2 mM dithiothreitol and 1X protease inhibitor cocktail (Roche, Mannheim, Germany), then sonicated using a Vibracell VCX130 Ultrasonic Cell Disrupter (Sonics & Materials Inc., Newtown, CT, USA) for 30 sec on ice. Protein concentration was determined using a Bicinchoninic Acid Protein Assay kit (Pierce Biotechnology, Inc., Rockford, IL, USA). Cell lysates (50 μg per sample) were separated by 10% SDS-PAGE and electrotransferred to nitrocellulose membranes. After blocking with 5% non-fat milk in 0.1% Tris-Buffered Saline with Tween 20, the membranes were incubated with primary monoclonal mouse anti-human CEP65 antibody (dilution, 1:500) overnight at 4°C, then reprobed with horseradish peroxidase-labeled goat anti-mouse secondary antibody (dilution 1:5,000; ZSGB-BIO, Beijing, China) for 45 min at room temperature. Signals were detected by an enhanced chemoluminescence system (Pierce Biotechnology, Inc.). Mouse monoclonal anti-human actin antibody (1:5,000; Sigma-Aldrich) was used to normalize loading.

### cDNA microarray analysis

Total RNA was isolated from the cells using TRIzol (Invitrogen Life Technologies). Poly (A)^+^ RNA was annealed to oligo(dT) and transcribed with avian myeloblastosis virus reverse transcriptase in the presence of Cy3-dUTP or Cy5-dUTP. Labeled cDNAs were co-hybridized (AGS-CEP65 vs. AGS; or AGS-CEP65 vs. AGS-MOCK) to a BiostarH-141s chip (Biostar Genechip Inc., Shanghai, China) containing cDNAs from >14,000 human genes. The washed slides were scanned using a ScanArray 4000 Array scanner (PerkinElmer, Inc., Waltham, MA, USA). Following background subtraction, filtering of inappropriate spots (including spots with an improper morphology and intensity/background ratio, and a small size) and global normalization, the signals were analyzed using GenePix Pro 3.0 software (Molecular Devices, LLC, Sunnyvale, CA, USA). Genes were considered to be downregulated or upregulated if the difference in the ratio was >2-fold.

### Semiquantitative RT-PCR

Total RNA (10 μg) was treated with DNase I (Pierce Biotechnology, Inc.) and reverse transcribed using a reverse transcription Kit (GoScript Reverse Transcription System, Promega Corporation). The genes of interest were PCR-amplified using a variable number of cycles (25–32 cycles) and 100 ng cDNA. The PCR conditions for each cycle were as follows: Denaturation at 95°C for 30 sec, annealing at 57°C for 30 sec and extension at 72°C for 60 sec. Glyceraldehyde 3-phosphate (*GAPDH*) was used as an internal control. The primers were as follows: *CEP65* forward, 5′-CCCTTTCTCAAC AGACTCATATGAA-3′ and reverse, 5′CAAGGCCCACACGCTCTC-3′; MMP2 forward, 5′-TGCAATACCTGAACACCTTC-3′ and reverse, 5′-AAGGTCCATAGCTCATCGTC-3′; *MMP9* forward, 5′-TCCTACTCTGCCTGCACCAC-3′ and reverse, 5′-ACAGGTCGAGTACTCCTTAC-3′; *VTN* forward, 5′-TCTGCTCTTACTACCCAGAGC-3′ and reverse, 5′-GACATCTCGGATGAGCTTGG-3′; *RAP* forward, 5′-TTAGGATCCATGGCGCCGCGGAGGGTCAG-3′ and reverse, 5′-AGGGAATTCAGAGTTCGTTGTGCCGAGC-3′; *TIMP-2* forward, 5′-TTTGCAATGCAGATGTAGTG-3′, and reverse 5′-TGGGTCCTCGATGTCGAGAAAC-3′; and *GAPDH* forward, 5′-ACCACAGTCCATGCCATCAC-3′ and reverse, 5′-TCCACCACCCTGTTGCTGTA-3′. The PCR products were visualized on 1.2% agarose gel containing 0.05 μg/ml ethidium bromide.

### Statistical analysis

The values are expressed as the mean ± standard deviation from at least three independent experiments in triplicate wells. SPSS 11.0 software (SPSS Inc., Chicago, IL, USA) was used to perform an analysis of variance and unpaired Student’s t-test. P<0.05 was considered to indicate a statistically significant difference.

## Results

### CEP65 promotes AGS cell growth and invasiveness in vitro

**Figure 1:**
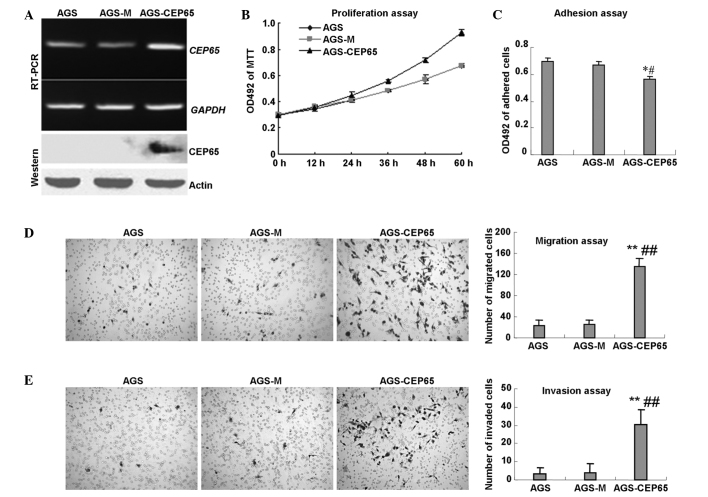
CEP65 promotes AGS cell growth and invasiveness *in vitro*. (A) Expression of CEP65 in transfected AGS cells, as detected by RT-PCR and western blot analysis. (B) CEP65 promotes the growth of AGS cells. An MTT assay was performed and proliferation curves were generated by measuring the optical density (OD) at 490 nm at the indicated time-points after plating. (C) CEP65 decreases the adhesion of the AGS cells to the Matrigel. Data represent the results from three independent determinations. (D) CEP65 promotes the migration of the AGS cells. Cells were allowed to migrate for 12 h after plating. (E) CEP65 promotes the invasion of the AGS cells. Cells were allowed to migrate for 16 h after plating. ^*^P<0.05 and ^**^P<0.01 vs. AGS; ^#^P<0.05 and ^##^P<0.01 vs. AGS-M. CEP65, cancer and embryo expression protein 65; GAPDH, glyceraldehyde 3-phosphate; AGS-M, AGS mock-transfected cells.

Although CEP65 overexpression has been reported in various cancerous tissue types (1), the endogenous levels of CEP65 in >30 cancer cell lines were undetectable on western blot analysis (data not shown). To overcome this limitation and examine the biological effects of CEP65 in cancer cells, the human full-length CEP65 was cloned (3) and then stably expressed in the AGS gastric cancer cell line (AGS-CEP65), using cells expressing the vector (AGS-M) and parental cells as controls. Western blot and RT-PCR assays verified the expression of exogenous CEP65 (Fig. 1A). In addition, an MTT assay was performed and CEP65 was found to significantly promote the growth of AGS cells (Fig. 1B). Furthermore, the adhesion ability of the AGS cells to Matrigel mimicking extracellular matrix (ECM) was found to be reduced by 16.2 and 18.8% in the AGS-CEP65 cells compared with the AGS-M and AGS cells, respectively (P<0.05; Fig. 1C). Next, cell migration and invasion assays were performed to investigate the invasiveness of the AGS-CEP65 cells. As shown in Fig. 1D, the motility was increased by 4.1- and 4.7-fold in the AGS-CEP65 cells compared with the AGS-M and AGS cells, respectively (P<0.01). The results of the invasion assay (Fig. 1E) revealed that the invasive capacity was also increased by 6.9- and 8.4-fold in the AGS-CEP65 cells compared with the AGS-M and AGS cells, respectively (P<0.01). No statistically significant differences were observed between AGS-M and AGS in the migration or invasion assays (P>0.05). These results indicated that CEP65 promoted cancer cell growth and invasiveness *in vitro*.

### CEP65 promotes BICR-H1 cell growth and metastasis in vivo

**Figure 2:**
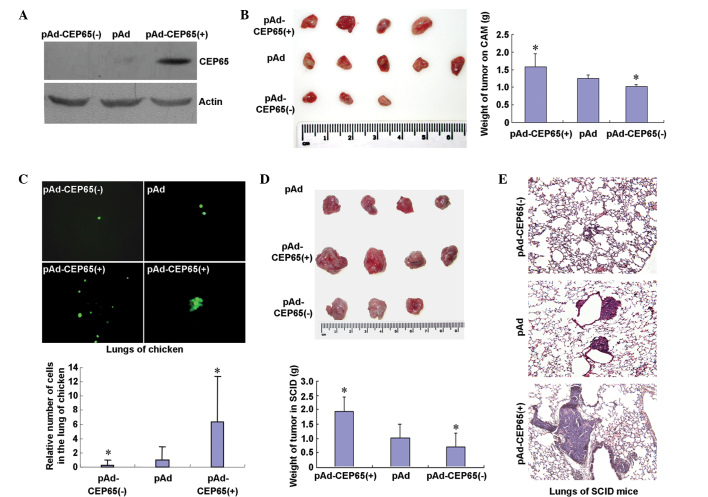
CEP65 promotes BICR-H1 cells growth and metastasis *in vivo*. (A) Expression of CEP65 in adenovirus-infected BICR-H1 cells, as detected by western blot analysis. (B) CEP65 promotes tumor formation on chick chorioallantoic membranes: Left image, macroscopic image of tumors; right image, graph summarizing the weight of the tumors. (C) CEP65 promotes BICR-H1 metastasis to the lungs of the chicken: Left, metastatic foci in the chicken lungs were detected using fluorescence microscopy; right, graph summarizing the numbers of metastatic foci. (D) CEP65 promotes tumor formation in SCID mice: Left, macroscopic image of tumors; graph summarizing the weight of the tumors. (E) CEP65 promotes BICR-H1 metastasis to the lungs of SCID mice, as detected by hematoxylin and eosin staining. ^*^P<0.05 vs. pAd. CEP65, cancer and embryo expression protein 65; SCID, severe combined immunodeficiency.

To validate the results from the *in vitro* assays, the effects of CEP65 on cancer cell growth and metastasis were further investigated *in vivo*. Due to the low capacity of AGS cells to form metastatic foci in animals, BICR-H1 cells were used in the present study; these are breast cancer cells with defined metastatic ability that have been used in a previous study (9). CEP65 was barely detectable in the BICR-H1 cells, using western blot analysis (Fig. 2A). Adenovirus-mediated transduction of antisense CEP65 [pAd-CEP65(−)] resulted in the complete elimination of CEP65 expression (Fig. 2A), while transduction of sense CEP65 [pAd-CEP65(+)] was found to strongly increase the CEP65 protein levels (Fig. 2A). Following inoculation of the plasmids on the chick CAMs for seven days, tumors were formed (Fig. 2B). The tumors were weighed, revealing that pAd-CEP65(+) increased tumor growth (P<0.05), whereas pAd-CEP65(−) decreased tumor growth (P<0.05) (Fig. 2B). Using fluorescence microscopy, the metastasis of the BICR-H1 cells to the chicken embryo lungs was increased by 5.3-fold in the cells infected with pAd-CEP65(+) as compared with the cells infected with pAd (P<0.05). Metastasis was found to be reduced by 76.2% in the cells infected with pAd-CEP65(−) as compared with the cells infected with pAd (P<0.05; Fig. 2C). Furthermore, infected BICR-H1 cells were transfected into the mammary fat pads of SCID mice and the CEP65 expression levels were found to be positively correlated with cell growth (Fig. 2D) and metastatic foci formation in the lungs (Fig. 2E). These results indicated that CEP65 promoted the growth and metastasis of the BICR-H1 cells *in vivo*.

### CEP65 induces MMP2 activity

**Figure 3:**
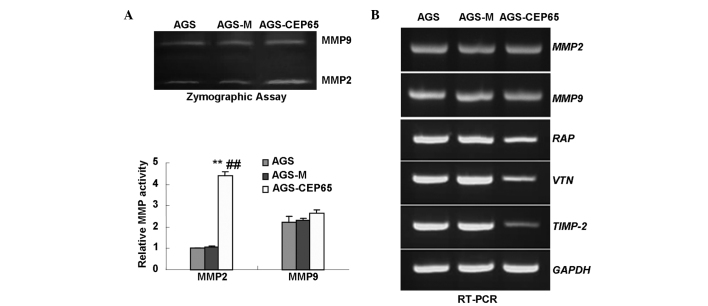
CEP65 promotes MMP2 activity and decreases expression levels of metastasis-related genes. (A) CEP65 promotes MMP2 activity. Serum-free conditioned media from indicated cells were subjected to zymographic assay. Following removal of the SDS, the gelatin-containing gel was incubated in developing buffer and then visualized by Coomassie blue staining (upper panel). Relative activities of MMP2 and MMP9 were calculated (lower panel). (B) Effect of CEP65 on gene expression, as detected by reverse transcription polymerase chain reaction. ^**^P<0.01 vs. AGS; ^##^P<0.01 vs. AGS-M. CEP65, cancer and embryo expression protein 65; MMP, matrix metalloproteinase; GAPDH, glyceraldehyde 3-phosphate; AGS-M, AGS mock-transfected cells.

The results of the present study suggested that CEP65 has a proinvasive function in cancer cells, while the mechanism underlying this role remains unclear. Since the degradation of the ECM by MMPs is essential in metastasis (11), the activities of MMP2 and MMP9 in the culture supernatant of AGS cells were investigated by zymographic assay. As shown in Fig. 3A, the collagen-degrading activity of MMP2 was higher in the AGS-CEP65 cell supernatant compared with the activity in the AGS and AGS-M cells (P<0.01), however, CEP65 did not exhibit a stimulatory effect on the activity of MMP9. The mRNA expression levels of MMP2 and MMP9 were not affected by CEP65 (Fig. 3B). Therefore, the increased MMP2 activity in the conditioned medium of AGS-CEP65 cells did not result from the upregulation of MMP2 expression.

### Expression of CEP65 in AGS cells results in altered gene expression

In order to understand the role of CEP65 in regulating cancer cell invasiveness, a microarray analysis was performed to screen genes with expression levels that were affected by CEP65 in AGS cells. The results revealed 39 candidate genes with >2-fold decreased expression levels and 28 genes with >2-fold increased expression levels in the AGS-CEP65 cells. Metastasis-associated genes, including *RAP, TIMP-2* and *VTN*, were among the downregulated genes. RT-PCR analysis confirmed the decreased expression levels of these three genes in the AGS-CEP65 cells (Fig. 3B).

## Discussion

Previous studies have demonstrated that 3H11-Ag/CEP65 is highly expressed in gastric cancer tissues (1,2). In other previous studies, 3H11-Ag/CEP65 was cloned from a cDNA library of MGC803 gastric cancer cells (3) and CEP65 was tagged with a red fluorescent protein (RFP), which was detected in the nucleus and cytoplasm of COS-7 cells (6). In addition, differential extraction has indicated that CEP65 is a potential DNA-binding protein and peripheral membrane protein associated with nuclear envelopment. The 150 amino acid C-terminal sequence of CEP65 may determine its subcellular localization (6). However, the biological role of CEP65 remains to be elucidated. In the present study, the stimulatory effects of CEP65 in the promotion of cancer cell growth and metastasis were identified through *in vitro* and *in vivo* experiments, revealing that CEP65 is a critical oncogene in tumor development.

CEP65 was found to decrease cell adhesion to Matrigel, which was correlated with the decreased mRNA expression level of VTN, as identified by microarray screening and RT-PCR analysis. *VTN* has been identified as a cell adhesion molecule promoting cell attachment (12–14), therefore, the deregulation of *VTN* expression by CEP65 may provide cancer cells with the capacity to detach from the ECM.

Consistent with enhanced invasiveness, CEP65 was demonstrated to promote MMP2 activity in a zymographic assay. However, CEP65 did not affect the mRNA expression of MMP2, indicating that the elevated MMP2 activity may be due to altered regulators upstream of *MMP2*. MMP2 belongs to a family of Zn^2+^-dependent proteolytic enzymes, which are associated with cancer cell invasion, growth, angiogenesis, inflammation and metastasis (11,15). These enzymes are counter-balanced by TIMPs, the endogenous antagonists of MMPs (16,17). The TIMP family consists of at least four distinct members, including TIMP-1, -2, -3 and -4 (16,17). TIMP-2 is a non-glycosylated protein, which can bind to various MMPs and antagonize their activity, exhibiting the highest inhibitory activity against MMP2 (16). A number of studies have reported TIMP-2-mediated inhibition of tumor growth and invasion (18–22), while lower levels of TIMP-2 in tumor samples have been associated with tumorigenesis (23). Blocking TIMP-2 activity with an anti-TIMP-2 antibody has been demonstrated to significantly induce DOV13 cell invasion due to increased vascular endothelial growth factor (VEGF) expression (24). Another study demonstrated that, in addition to its ability to block the action of MMPs, TIMP-2 can also modulate tumor-host interactions, angiogenesis, tumor growth, cell differentiation (through the regulation of cell-cycle regulatory proteins), disruption of VEGF signaling and inhibition of the mitogenic response of human microvascular endothelial cells to growth factors (16), which are MMP-independent effects. Further investigation is required to establish whether CEP65 also functions through the aforementioned pathways.

The present study revealed that CEP65 downregulated RAP mRNA expression. RAP, a 39-kDa polypeptide that is predominantly located in the endoplasmic reticulum, was identified as a molecular chaperone that is co-purified with low density lipoprotein receptor-related protein 1 (LRP1) (25–28). LRP1 is involved in the catabolism of proteinases by facilitated internalization (29). When LRP1 activity is reduced, the internalization process is impaired and the activity of the proteinases is increased (30). These changes in activity initiate a variety of signaling events and promote cell migration and invasion (31). Further investigation is required to establish whether the downregulation of RAP induced by CEP65 is involved in enhanced cell migration and invasion.

Another critical issue that requires investigation is how CEP65 regulates mRNA expression of *TIMP-2*, *VTN* and *RAP*. CEP65 has been identified to contain eight coiled-coil domains (3), which are involved in the mediation of protein-protein interactions (32). To date, the spectrum of CEP65-binding proteins is unclear, while several other centrosomal proteins have been found to be involved in the regulation of gene transcription (5,33). For instance, NPHP6/CEP290 is physically associated with activating transcription factor 4 (ATF4), which may synergistically regulate renal development (5). Considering the high homology between CEP65 and NPHP6/CEP290, investigation of the possible involvement of CEP65 in transcriptional regulation through ATF4 may be useful.

In conclusion, the present study demonstrated that CEP65 enhanced cancer cell growth, migration, invasion and metastasis, which was correlated with the decreased expression levels of *TIMP-2*, *VTN* and *RAP*. These oncogenic roles also indicated that CEP65 may be a potential target for cancer therapy.
